# Interoceptive Awareness Mediates Effects of Sleep Disturbance on Pain Outcomes in Chronic Pain Patients

**DOI:** 10.1089/imr.2023.0019

**Published:** 2024-05-30

**Authors:** Dana Dharmakaya Colgan, Natasha Parman

**Affiliations:** 1 Oregon Center for Complementary and Alternative Medicine in Neurological Disorders, OR Health and Science University, Portland, OR, USA; 2 National University of Natural Medicine, Helfgott Research Institute, Portland, OR, USA; 3 School of Nursing, University of Washington, Seattle, WA, USA

**Keywords:** interoceptive awareness, sleep, pain, predictors, mediation

## Abstract

**Background:**

Sleep disorders are common in adults with chronic pain and are multifactorial. Recent evidence suggest sleep disturbance may affect interoceptive awareness (IA) in healthy adults. Less is known about the relationship between IA, sleep, and pain in adults with chronic pain.

**Purpose:**

This cross-sectional study investigated the relationships between perceived sleep disruption (PROMIS Sleep), pain interference (PROMIS Pain Interference Short Form), pain intensity (PROMIS Pain Intensity Scale), and IA (Multidimensional Interoceptive Awareness Scale-2 [MAIA-2]), and subsequently, delineated potential direct and indirect links among constructs.

**Methods:**

Online surveys were administered to 301 individuals with chronic pain. Strategic sampling targeted respondents to reflect the 2010 census. Pearson’s correlation characterized the overall relationship between variables. Hierarchical multiple regression analyses were conducted to investigate direct links between sleep disturbance, pain outcomes, and IA. Path analyses assessed mediational effects of IA on the relationship between sleep disturbance and pain outcomes.

**Results:**

Increased perceptions of sleep disturbance predicted increased pain interference (*p* ≤ 0.001, *β* = 0.42), increased pain intensity (*p* ≤ 0.001, *β* = 0.36), and reduced IA (*p* ≤ 0.001, *β* = −0.38) when controlling for pain duration. IA partially mediated the relationship between sleep disturbance and pain interference (point estimate = 0.16; 95% bootstrap confidence interval [CI] = 0.08–0.24) and between sleep disturbance and pain intensity (point estimate = 0.06; 95% bootstrap CI = 0.02–0.09).

**Conclusions:**

Findings show increased sleep disturbance predicted increased pain interference and increased pain intensity and reduced IA, and that IA partially mediated the relationship between sleep disturbance and pain characteristics. Findings support future research to explore causal relationships of these variables in longitudinal studies.

## Introduction

Chronic pain affects over 100 million Americans, costs the United States approximately $635 billion per year,^1^ and can negatively impact physical, emotional, social, and cognitive functioning in people affected.^2^ Numerous cross-sectional studies have shown a high comorbidity between sleep impairment and chronic pain. A recent meta-analysis estimated a pooled prevalence of sleep disorders in 44% of adult chronic pain patients and reported that patients with chronic pain had worse measures for sleep onset latency and efficiency, time awake after onset, and recurrent awakenings when compared to controls.^3^

The association between poor sleep quality and pain is well-established; however, the directionality of this relationship has been debated. Previously, the relationship was thought to be bidirectional: sleep disturbance exacerbated pain and pain contributed to sleep disturbance. Recent qualitative analyses of longitudinal and microlongitudinal time studies, however, suggest a stronger and more consistent unidirectional effect of sleep disturbance causing pain exacerbation, particularly in experimental and acute pain models,^4–6^ and demonstrated among samples of pediatric and adult chronic pain populations,^7–9^ as well as the general population.^10^ Furthermore, good sleep quality has been shown to predict partial resolution of pain. Pain, however, does not seem to predict persistence versus remission of insomnia.^11^

Results from cross-sectional and longitudinal brain imaging studies suggest that the chronification of pain is correlated with a shift away from acute pain circuits to the engagement of emotion circuits.^12–14^ Thus, enhancing self-regulation of arising sensory and affective experiences is now considered a candidate mechanism in promoting long-lasting reductions in pain and corresponding comorbidities.^15,16^ In parallel, growing evidence suggest that sleep may impair processes of interoception.^17^ Interoception is the process by which the central nervous system receives, appraises, and responds to internal bodily signals,^18^ and is an essential process to maintain physical and emotional homeostasis.^19,20^ Processes of interoception are largely unconscious; however, a conscious dimension of interoception, coined interoceptive awareness (IA), includes the subjective perception of bodily sensations, and subsequently, the cognitive and emotional reactions to those sensations.^21^

Importantly, the quality of an individual’s attention to bodily sensations, as well as the secondary appraisals of sensations, have both been reported to be of prognostic key importance within chronic pain populations.^22,23^ Individuals with increased adaptative IA are more likely to have an attentional stability on bodily sensations. Importantly, this quality of attention is decoupled from habitual cognitive-affective reactivity. This type of attention may provide more precision of the present-moment characteristics of physical sensations, thereby informing more skillful decision-making to maintain homeostasis.^18,21,22,24^ Adaptive IA has been associated with favorable outcomes for chronic pain (For extensive review, see Mehling et al., 2009).^25^ Specifically, adaptive IA has been found to be associated with decreased pain unpleasantness^26^ and symptoms related to central sensitization,^27,28^ increased pain tolerance,^29^ increased parasympathetic activation,^29^ and increased descending pain inhibition.^30^

Impaired, or maladaptive, IA is understood as a quality of attention that involves avoidance, ignoring, or suppression of bodily sensation. It is also described as a tendency to appraise bodily sensations as dangerous or threatening. This quality of appraisal is thought to facilitate central pain processing through the activation of limbic system, which, in turn, contributes to and maintains pain.^31,32^

Previous research suggests that sleep may influence IA, but the research is scant. Three cross-sectional studies of generally healthy adults found sleep disturbance was a significant predictor of IA.^33–35^ The Sleep and Pain Diathesis (SAPD) model^36^ locates sleep impairment upstream as an etiological factor and also a “driving force” that activates a cognitive feedback loop to maintain a broad range of pain symptoms. Specifically, the SAPD model proposes that sleep disruption, in those most sensitive to pain, may initiate a cascade of symptoms, including central and autonomic nervous systems and inflammatory responses, resulting in increased pain and fatigue. Associated symptoms are then perpetuated and aggravated by reduced attentional and affective regulation of pain signals (i.e., IA), which increase perceptions of threat, ultimately manifesting more pain.

To our knowledge, no study has investigated the relationship between sleep, pain, and IA within a chronic pain population. Successful management of chronic pain requires a better understanding of how sleep contributes to or alleviates the pain experience. Therefore, the primary aims were to investigate relationships between self-reported sleep disturbance, IA, and pain outcomes (i.e., pain interference and pain intensity), and subsequently, delineate potential indirect links among constructs.

### Aims and hypotheses

The primary aim was to assess if sleep disruption predicts patient-reported pain intensity, pain interference, and reduced IA. The second aim was to evaluate IA as a mediator of the relationship between sleep disturbance and these pain outcomes. We hypothesized the following:

1. Increased sleep disturbance would significantly predict increased patient-reported pain intensity and interference.
2. Increased sleep disturbance would significantly predict reduced adaptive IA.
3. Adaptive IA would mediate the relationship between sleep disturbance and patient-reported pain intensity and interference.

## Methods

In this cross-sectional study, we conducted secondary analyses using a subsection of a larger survey battery designed to examine the psychometric properties of a new self-report survey instrument (for details of methods section, review). The study was approved by the Oregon Health & Science University’s institutional review board and all participants provided informed consent before participating. Qualtrics (Version 2020. https://www.qualtrics.com) was contracted to recruit a randomly selected, US, USA national sample. Strategic quota sampling was used to target respondents as to reflect the 2010 census stratified in domains of race, ethnicity, geographic location, age, sex identified at birth, education, and income. Eligible participants were English-speaking adults, were 18–70 years of age, had internet access, and reported chronic pain, as defined by the National Institute of Health Task Force for Research Standards for Chronic Pain.^37^ Exclusion criteria included the endorsement of being currently involved with a workers’ compensation claim or currently undergoing a disability application or claim.

Qualtrics recruited 2,743 participants to take the survey. Of these individuals, 301 participants met inclusionary criteria, consented to the study, and completed an online battery of validated questionnaires. Questionnaires were presented in a randomized order to reduce ordering effects. Qualtrics used several different algorithms to identify potential violations such as copying or pasting answers, falsification of data entry, or altering the order of responses.

### Measures

Demographic information collected included sex identified at birth, gender, race/ethnicity, age, education, employment, income, relationship status, chronic pain duration, and pain location.

The PROMIS Pain Interference Short Form (4a)^38^ is a four-item scale that measures the degree to which pain interferes in daily living. Items were rated on a 5-point Likert-type scale ranging from 1 (*Not at all*) to 5 (*Very much*). The questions ask how much pain has interfered with day-to-day activities, work around the home, the ability to participate in social activities, and household chores in the past seven days. A total score was calculated by summing scores across all items, with higher scores indicating more pain interference. Scores ranged from 4 to 20. The measure demonstrates excellent reliability (α = 0.90).

The PROMIS Numeric Rating Scale—Pain Intensity^38^ is a one-item scale that assesses average pain intensity over the last seven days using a numerical rating scale of 0 (*No pain*) to 10 (*Worse imaginable pain*). Higher scores indicate greater pain intensity.

The PROMIS Sleep Disturbance Short Form (4a)^38^ is a four-item scale that measures sleep disturbance in the past 7 days. The first item assesses sleep quality, with response optio**ns** ranging from 1 (*Very **g**ood*) to 5 (*Very **p**oor*). The remaining items include the following: “My sleep was refreshing…” with response options ranging from 1 (*Very much*) to 5 (*Not at all*); and “I had a problem with my sleep…” and “I had difficulty falling sleep…” with response options ranging from 1 (*Not at all*) to 5 (*Very much*). Higher scores indicate more disrupted sleep (e.g., lower quality), with scores ranging from 4 to 20. The measure demonstrates good reliability (α = 0.79).

The Multidimensional Assessment of Interoceptive Awareness (MAIA-2)^39^ scale is a 37-item instrument that assesses awareness of bodily sensations in eight subscales, organized into five domains. Based on previous research^12–14,27,28^ and our interest in the SAPD model,^36^ which highlights reduced emotional and attentional regulation of pain signals, we selected the Emotional and Attentional Responses to Bodily Sensations domain, which includes the Not-Worrying and the Not-Distracting subscales. The Not-Worrying subscale assesses the tendency not to worry or experience emotional distress with sensations of pain or discomfort. Items include the following: “When I feel physical pain, I become upset”; “I start to worry that something is wrong if I feel any discomfort”; “I can notice an unpleasant body sensation without worrying about it”; “I can stay calm and not worry when I have feelings of discomfort or pain”; and “When I am in discomfort or pain I can’t get it out of my mind.” The Not-Distracting subscale assesses the tendency to not ignore or distract oneself from uncomfortable body sensations such as pain. Items include the following: “I ignore physical tension or discomfort until they become more severe”; “I distract myself from sensations of discomfort”; “When I feel pain or discomfort, I try to power through it”; “I try to ignore pain”; “I push feelings of discomfort away by focusing on something”; and “When I feel unpleasant body sensations, I occupy myself with something else so I don’t have to feel.” Items are rated on a 6-point Likert-type scale ranging from 0 to 5, with higher scores equating to more awareness of bodily sensation. For the subscales used, total scores range from 0 to 55. These factors demonstrated acceptable to excellent reliability (Not-Worrying, α = 0.64; Not Distracting, α = 0.84).

### Statistical analyses

All statistical analyses were performed using IBM SPSS for Windows version 26.0^40^ at an alpha level of 0.05. All variables were examined to evaluate data compliance with parametric analysis assumptions. Pearson’s correlations were conducted to characterize the overall relationship between variables. We employed hierarchical multiple regression analyses to investigate if sleep disturbance predicts pain interference and pain intensity (Hypothesis 1). Two path analyses using the PROCESS procedure for SPSS^41^ were conducted to assess: IA as a mediator in the path between sleep disturbance and pain outcomes (Hypothesis 2). For all mediation models, we employed a bootstrapping method to compute an estimate of the indirect effects. Bootstrapping is a nonparametric resampling method that bypasses assumptions of normality common to traditional tests of mediation, and is, thus, more powerful.^41,42^ In particular, 5,000 samples of the original size were taken from the obtained data (with replacement after each specific number was selected), and indirect effects were calculated in each sample. Mean indirect effect computed over each of these 5,000 samples was used to compute the point estimate. The bias corrected and accelerated 95% confidence intervals (CIs; i.e., with *z* score-based corrections for bias due to the underlying distribution) were then examined, and if these intervals did not contain 0, the point estimate of the indirect effect was considered significant. For all regressions, covariates were added to the model before the predictor variable if their *p*-value was less than 0.10. The alpha level was set at 0.05 (two tailed) for all analyses. Sample size was based on statistical power analysis conducted using G*Power software tool.^43^ Using the number of predictors as three, a medium effect size level (0.15), a moderate significance level (α = 0.05), and a power requirement of 0.95, the minimum required sample size was 119.

## Results

Participant demographics (*n* = 301) are reported in Table 1. All surveys were completed remotely online in a single administration session lasting an average of 29 min. The mean age was 45 years (*SD* = 15.5) and 48% identified as female. All participants in the sample reported moderate pain intensity (*M* = 5.17; *SD* = 2.16) on a 0–10 Numerical Rating Scale. Pain duration ranged from 3 months to over ten years. Seventy-nine percent of the sample reported pain in the neck or back region, 5% in the head (i.e., migraines), 2% in pelvic region, and 15% in another region. Men and women did not differ in reported pain outcomes or variables of interest. Bivariate correlations revealed sleep disturbance was significantly associated with pain intensity and interference and significantly and negatively associated with adaptive IA. Adaptive IA was also significantly and negatively associated with pain outcomes. Duration of pain was significantly correlated with sleep disturbance and pain intensity (Table 2).

### Regression analyses

Consistent with hypothesis 1, when controlling for duration of pain, perceptions of sleep disturbance predicted pain interference (*R*^2^ = 0.21, *F*[2,298] = 25.70, *p* ≤ 0.001, *β* = 0.42) and pain intensity (*R*^2^ = 0.15, *F*[1,299] = 51.80, *p* ≤ 0.001, *β* = 0.36), such that as sleep disturbance increased by one unit, pain interference increased by 0.69 units and pain intensity increased by 0.31 units (Table 3). A third regression analyses revealed that when controlling for duration of pain, reduced IA predicted increased pain intensity (*R*^2^ = 0.13, *F*[2,298] = 21.76, *p* ≤ 0.001, *β* = −0.28) and pain interference (*R*^2^ = 0.15, *F*[2,298] = 26.51, *p* ≤ 0.001, *β* = −0.38), such that for every one unit decrease in IA , pain intensity was predicted to increase by 0.3 units and pain interference was predicted to increase by 0.7 units. Finally, when controlling for duration of pain, perceptions of sleep disturbance predicted reduced IA (*R*^2^ = 0.11, *F*[2,298] = 12.24 *p* ≤ .001, *β* = −0.38), such that as sleep disturbance increased by one unit, IA decreased by 1.3 units (Table 4).

### Path analyses

To assess hypothesis 2, we conducted two path analyses in which sleep disturbance was entered as the independent variable, pain interference or pain intensity as the dependent variable, and IA was entered as the mediator. Results from the first path analysis revealed that IA partially mediated the relationship between sleep disturbance and pain interference (point estimate = 0.16; 95% bootstrap CI = 0.08–0.24; Table 5). Results from the second path analysis revealed IA partially mediated the relationship between sleep disturbance and pain intensity (point estimate = 0.06; 95% bootstrap CI = 0.02–0.09; Table 6).

## Discussion

To our knowledge, this is the first study to report on the potential mediational effects of IA in the path between sleep disturbance and pain outcomes in a sample of chronic pain participants. Congruent with previous studies among chronic pain populations,^3,4,27,28^ results indicated that perceptions of increased sleep disturbance predicted increased pain interference and pain intensity and reduced adaptive IA. Reduced adaptive IA predicted increased pain intensity and interference. Effects on IA were found to partially mediate the relationship between sleep disturbance and pain interference and sleep disturbance and pain intensity. Current findings supported our hypotheses and provide preliminary evidence that within chronic pain populations, poor sleep may negatively impact processes of IA, which then increases pain intensity and pain interference.

Findings are also congruent with results from studies among non-pain populations, which investigated effects of poor sleep on IA. For example, Ewing et al.^30^ reported that among individuals with mental health conditions, poor sleep quality was associated with reduced IA, as measured by a behavioral marker. Specifically, among individuals with diagnoses of depression and/or anxiety, poor sleep quality was associated with lower measures of interoceptive accuracy and reduced correspondence between perceived ability and actual performance measures. Authors concluded that the perceived quality of sleep directly influenced interoceptive processing, and the influence was, in part, explained by the unfavorable effect of sleep on attentional and cognitive processes.

Finally, results align with the SAPD model.^36^ In support of the SAPD theory, poor cognitive and affective regulation is considered one of the most important mediators in the sleep and pain relationship. Poor cognitive–affective regulation is thought to increase arousal and hypervigilance to pain, causing sensitization to pain, avoidance, and functional disability. A systematic review of studies evaluating path mediation found negative mood and affect, depression and anxiety, and pain helplessness were identified as mediators on the path between sleep impairment and pain intensity.^44^ Our findings uniquely identify mediating effects of IA (i.e., affective and cognitive regulation *specifically* as it relates to a person’s response to internal bodily sensations) on the path between sleep and pain, and align with previous studies that suggest enhancing self-regulation of arising sensory and affective experiences is an important candidate mechanism in promoting long-lasting reductions in pain and corresponding comorbidities.^12–14^ Understanding the relationship between IA, sleep, and pain outcomes may potentially inform more effective and personalized treatment approaches for patients with chronic pain, and other disorders.

Importantly, a notable strength of this study is the diverse sample. By using strategic quota sampling, we were able to target respondents as to reflect the 2010 census in the domains of race, ethnicity, geographic location, age, sex identified at birth, education, and income. This improves the generalizability of our results.

Important limitations of this study must also be noted. Causality could not be determined in this study due to the cross-sectional design. Longitudinal data are favored for testing associations between changes in latent traits. Although results from cross-sectional mediation analyses can inform theory when viewed as a type of variance partitioning, rather than a proxy for longitudinal relations, and in this way, mediation analyses can be valuable, even if none of the variables include a temporal dimension. Another limitation is the lack of biological or behavioral markers of sleep or pain due to the cross-sectional study design.

Future studies may benefit from utilizing microlongitudinal designs using ecological momentary assessment, to allow within-person variations to be studied, as well as adjustment for person-level variables. Studies using brain imaging (e.g., fMRI), electrophysiology, or other imaging techniques are needed to increase our understanding of the neural contributions underlying the sleep and pain relationship. Finally, novel modeling techniques will be needed to thoroughly assess potential relationships between sleep and pain phenotypes. Machine learning, for instance, is a mathematical approach that can help to identify patterns or clusters in variables to characterize phenotypes and observe more precise characteristics of vulnerability among patients.

In sum, this study provides a unique contribution to the pain literature by investigating the mediating effects of IA on sleep in a sample of people with chronic pain. Our findings demonstrate that perceptions of sleep disturbances predicted reduced IA, and IA partially mediated the relationship between sleep disturbance and both pain interference and pain intensity. Understanding the relationship between IA, sleep, and pain outcomes may potentially inform more effective and personalized treatment approaches for patients with chronic pain. For example, health care providers may design and deliver interventions specifically aimed to improve IA or reduce sleep disturbances. This study may inform future research to investigate protective factors that may buffer or mitigate the sleep–pain cycle.

## Figures and Tables

**Table 1.** Participant Demographics (*n* = 301)

Measure	M	SD
Age	45	15.5
	*n*	%
Sex at Birth		
Male	154	51
Female	144	48
Intersex	3	>1
Race/Ethnicity		
Hispanic/Latinx/Spanish	53	17
Black	37	12
Asian	13	5
Native American	13	5
White	185	61
Education		
Less than High school	16	5
High school or equivalent	109	36
Some College	62	21
2-yr associate degree	22	7
4-yr bachelor’s degree	51	17
Master’s degree	34	11
Doctoral degree	6	2
Professional degree	1	.3
Employment		
Employed	144	48
Temporarily laid off/Unemployed	43	14
Retired	52	17
Disabled	34	11
Other	28	19
Income		
Less than 30,000	67	22
30,000–50,000	55	18
50,000–70,000	56	18
80,000–100,000	24	8
100,000–150,000	80	27
>150,000	19	6
Relationship Status		
Married	146	49
Widowed	9	3
Divorce/Separated	50	17
Never Married	96	32
Chronic Pain Duration		
3–6 months	30	10
6–12 months	40	13
1–3 years	68	23
3–5 years	54	18
5–10 years	40	14
More than 10 years	69	23
Pain Location		
Neck and Back Pain	236	78
Migraines	15	5
Hip/Pelvis	5	2
Other	45	15

**Table 2.** Mean, Standard Deviation, and Zero-Order Correlation Matrix of Outcome Variables (*n* = 301)

Variable	M	SD	2	3	4	5	6
1. Sleep Disturbance^a^	11.76	2.53	*0.43* **	*0.38* **	−0.39**	−*0.05*	*0.15* *
2. Pain Interference^b^	10.51	4.10		0.73**	−0.38**	0.10	0.11
3. Pain Intensity^c^	5.17	2.16			−0.31**	0.06	0.22*
4. Interoceptive Awareness^d^	39.14	8.56				−0.07	−0.11
5. Sex at Birth							0.10
6. Duration of Pain	5.8 years	1.63					

a PROMIS Sleep Disturbance Short Form (4a).

b PROMIS Pain Interference Short Form (4a).

c PROMIS Numeric Rating Scale—Pain Intensity.

d Multidimensional Assessment of Interoceptive Awareness—Version 2, subscales Not-Distracting and Not-Worrying.

* *p* < 0.05,

** *p* < 0.01.

**Table 3.** Evaluating Effects of Sleep Quality on Pain Outcomes Using Linear Regression Analysis (*n* = 301)

	B (SE)	*β*	*p*
*Pain Interference* ^a^			
Step One:			
Pain Duration	0.27 (0.14)	0.11	<0.06
Step Two:			
Sleep Disturbance^b^	0.69 (0.07)	0.42	<0.001
*Pain Intensity* ^c^			
Step One:			
Pain Duration	0.28 (0.07)	0.22	<0.001
Step Two:			
Sleep Disturbance^b^	0.31 (0.05)	0.36	<0.001

a PROMIS Pain Interference Short Form (4a).

b PROMIS Sleep Disturbance Short Form (4a).

c PROMIS Numeric Rating Scale—Pain Intensity.

**Table 4.** Evaluating Effects of Sleep Disturbance on Interoceptive Awareness Using Linear Regression Analysis (*n* = 301)

	B (SE)	*β*	*p*
Step One:			
Pain Duration	−0.56 (.30)	−0.11	0.07
Step Two:			
Sleep Disturbance^a^	−1.3 (.18)	−0.38	>0.001

a PROMIS Sleep Disturbance Short Form (4a).

**Table 5.** Mediation Effects of Interoceptive Awareness on the Relationship between Sleep Disturbance^a^ and Pain Interference^b^

Effect	b	95% CI	
		Lower	Upper
Total	0.68	0.52	0.85
Direct	0.53	0.35	0.70
Indirect (mediation)	0.16	0.08	0.24

a PROMIS Sleep Disturbance Short Form (4a).

b PROMIS Pain Interference Scale.

**Table 6.** Mediation Effects of Interoceptive Awareness^a^ on the Relationship between Sleep Disturbance^b^ and Pain Intensity^c^

Effect	b	95% CI	
		Lower	Upper
Total	0.31	0.22	0.39
Direct	0.25	0.15	0.34
Indirect (mediation)	0.06	0.02	0.09

a Multidimensional Interoceptive Awareness Scale.

b PROMIS Sleep Disturbance Short Form (4a).

c PROMIS Pain Intensity Scale.
